# Assessment of 3-month changes in bone microstructure under anti-TNFα therapy in patients with rheumatoid arthritis using high-resolution peripheral quantitative computed tomography (HR-pQCT)

**DOI:** 10.1186/s13075-017-1430-x

**Published:** 2017-10-04

**Authors:** Tomohiro Shimizu, Hyo Jin Choi, Ursula Heilmeier, Matthew Tanaka, Andrew J. Burghardt, Jingshan Gong, Nattagan Chanchek, Thomas M. Link, Jonathan Graf, John B. Imboden, Xiaojuan Li

**Affiliations:** 1 Department of Radiology & Biomedical Imaging, Musculoskeletal Quantitative Imaging Research, University of California San Francisco, 185 Berry Street, Suite 350, San Francisco, CA 94107 USA; 2 Department of Orthopedic Surgery, Graduate School of Medicine, Hokkaido University, Sapporo, Japan; 3 Department of Internal Medicine, Division of Rheumatology, Gachon University Gil Hospital, Incheon, Korea; 4 Department of Medicine, Division of Rheumatology, University of California San Francisco, San Francisco, California USA

**Keywords:** Rheumatoid arthritis, Anti-tumor necrosis factor alpha, High-resolution peripheral quantitative computed tomography, Bone erosion

## Abstract

**Background:**

Although one study showed minimal progression of erosions in patients with rheumatoid arthritis (RA) one year after TNFα inhibition therapy, no studies have investigated very early bone changes after initiation of anti-TNFα treatment. We investigated the effects of 3-month anti-TNFα treatment on bone erosion progression and bone microarchitecture in RA patients using high-resolution peripheral quantitative computed tomography (HR-pQCT).

**Methods:**

Patients with RA (*n* = 27) (17 in the anti-TNFα and 10 in the MTX-only group) underwent assessment of disease activity score in 28 joints (DAS-28), radiographs, 3-T magnetic resonance imaging (MRI) and HR-pQCT of metacarpophalangeal and wrist joints at baseline and 3 months. HR-pQCT-derived erosion volume, joint volume/width and bone microarchitecture were computed and joint destruction was assessed using Sharp and RAMRIS scorings on radiographs and MRI, respectively.

**Results:**

Overall, 73 erosions were identified by HR-pQCT at baseline. Over 3 months, the anti-TNFα group had decreased mean erosion volume; increased erosion volume was observed in one clinical non-responder. The MTX-only group in contrast, trended toward increasing erosion volume despite low disease activity. In the anti-TNFα group, joint-space width and volume of MCP joints decreased significantly and was positively correlated with erosion volume changes (*R* ^2^ = 0.311, *p* = 0.013; *R* ^2^ = 0.527, *p* = 0.003, respectively). In addition, erosion volume changes were significantly negatively correlated with changes in trabecular bone mineral density (*R* ^2^ = 0.353, *p* = 0.020) in this group. We observed significant correlation between percentage change in erosion volume and change in DAS-28 erythrocyte sedimentation rate and C-reactive protein CRP scores (*R* ^2^ = 0.558, *p* < 0.001; *R* ^2^ = 0.745, *p* < 0.001, respectively) in all patients.

**Conclusions:**

Using HR-pQCT, our data suggest that anti-TNFα treatment prevents erosion progression and deterioration of bone microarchitecture within the first 3 months of treatment, one patient not responding to treatment, had significant progression of bone erosions within this short time period. Patients with low disease activity scores (<3.2) can have continuous HR-pQCT-detectable progression of erosive disease with MTX treatment only. HR-pQCT can be a sensitive, powerful tool to quantify bone changes and monitor RA treatment short term (such as 3 months).

## Background

Rheumatoid arthritis (RA) is progressive chronic inflammatory arthritis characterized by bone and cartilage erosions and joint damage [1]. Bone erosions are usually irreversible and may occur in the first few months of RA onset [2]. The onset of bone erosions reflects a more severe course of RA, and is associated with a poor quality of life and increased mortality [3].

Tumor necrosis factor alpha (TNFα) is a proinflammatory cytokine involved in the pathogenesis of RA. It induces the production and release of other proinflammatory cytokines (IL-1, IL-6, IL-8), and also stimulates osteoclast activation in bone, eventually leading to bone erosions [3]. Several inhibitors of TNFα are currently available on the market and have proven to be a useful strategy to suppress inflammation in patients with RA. Thus, anti-TNFα treatments have been shown to prevent erosion progression compared to a treatment with methotrexate (MTX) alone [4–7]. In those studies, either the radiography-based modified Sharp scores [4–7] or magnetic resonance imaging (MRI) was used to determine erosion progression [8, 9].

High-resolution peripheral quantitative computed tomography (HR-pQCT), is a very sensitive imaging tool to detect bone erosions [10] and to evaluate cortical and trabecular bone mineral density (BMD) and microstructure in RA [11]. It has also been applied in studies of psoriatic arthritis [12] and hand osteoarthritis [13]. As first-generation HR-pQCT scanners allow for very high image resolution with an isotropic voxel size of 80 μm, HR-pQCT has also evolved as a very valuable imaging tool to detect bone erosions and to evaluate cortical and trabecular BMD and microstructure. Using conventional multidetector computed tomography (CT) with a voxel size to 400 μm × 400 μm × 400 μm, Moller-Dohn et al. [14] identified minimal erosive progression one year after TNFα inhibition therapy in patients with RA. However, no studies so far have used HR-pQCT to examine the very early changes in bone microarchitecture (erosion and bone density and structures) after anti-TNFα treatment.

Thus, the goal of our study was to employ HR-pQCT to characterize the bone microstructural and erosive changes in patients with low disease activity on MTX over 3 months and in patients with high disease activity 3 months after initiation of anti-TNFα therapy. We hypothesized that anti-TNFα treatment would decrease the progression of bone erosions in RA as detectable using HR-pQCT.

## Methods

### Subjects

We prospectively enrolled patients in our RA cohort from March 2014 to October 2016. All patients were 18 years of age or older and fulfilled the 2010 American College of Rheumatology/European League Against Rheumatism (EULAR) classification criteria for RA [15]. Concurrent use of prednisone was permitted in doses ≤ 10 mg/day. Two groups of patients were recruited. Inclusion criteria for patients in the methotrexate (MTX) only treatment group (MTX-only group) were low disease activity with a disease activity score in 28 joints (DAS-28) ≤3.2 [16] during the last 2 months prior to the baseline visit; MTX at a stable dose for ≥ 8 weeks; no biologic therapy during the previous 6 months; and no anticipated biologic therapy for the next year. Inclusion criteria for patients in the combined MTX and anti-TNFα treatment group (anti-TNFα group) were moderate to severe RA with DAS-28 > 3.2; these patients were scheduled to initiate anti-TNFα (certolizumab) in addition to an ongoing MTX regimen. DAS scores were used to assess clinical response from baseline to 3 months. HR-pQCT was performed at baseline (prior to anti-TNFα initiation in the anti-TNFα group) and after 3 months. DAS scores were obtained by board-certified rheumatologists (JBI and JG) on the same day of imaging. Serum samples were collected to measure C-reactive protein (CRP) and erythrocyte sedimentation rate (ESR) at baseline and 3-month follow up. The Institutional Review Board (IRB) for Human Research approved this study design (IRB# 12-10418) and written informed consent was obtained from all subjects prior to participating in the study.

### Imaging studies

#### HR-pQCT

**Fig. 1:**
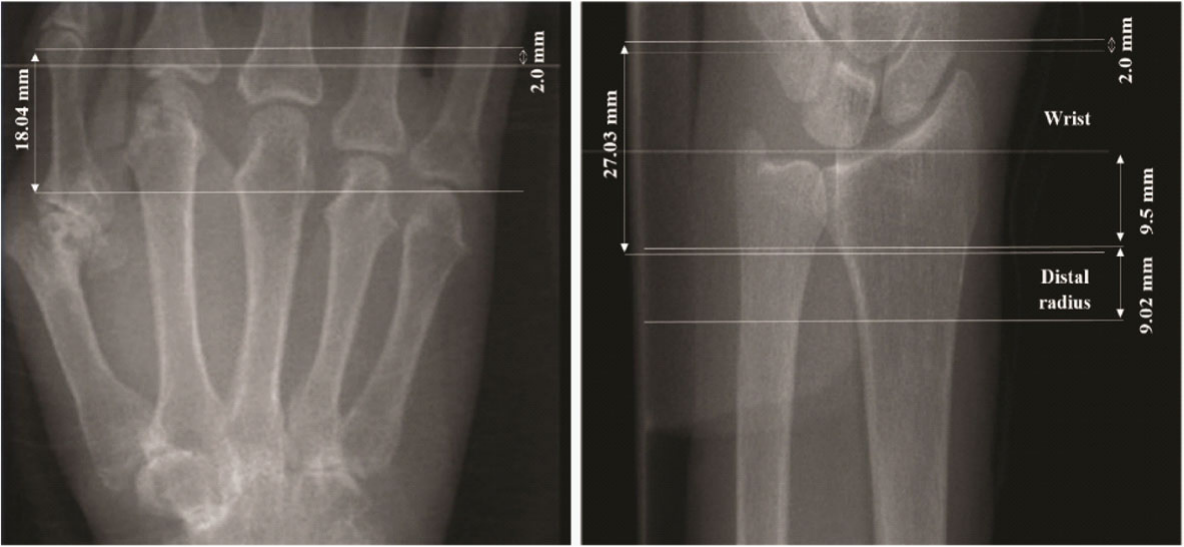
Scout film of metacarpophalangeal and wrist joints and distal radius on high-resolution peripheral quantitative images

All subjects were imaged with a first-generation HR-pQCT system (XtremeCT, Scanco Medical AG, Bruttisellen, Switzerland). HR-pQCT imaging was performed at the metacarpophalangeal (MCP) and wrist joints and distal radius of the dominant hand [17]. The distal radius scan was carried out in a manufacturer-provided forearm cast using the default imaging protocol [18], which covered 9.02 mm (110 slices) starting 9.5 mm distal to the mid-point of the radiocarpal joint surface of the radial head (Fig. 1). For the MCP and wrist joint acquisition, the patient’s forearm was immobilized in a palm-down orientation inside a custom carbon-fiber holder fit with an ergonomic thermoplastic molding. The MCP scan covered 18.04 mm (220 slices). The first slice was located 2 mm distal to the reference line position. The reference line was placed at the distal margin of the phalangeal base of the distal-most joint (MCP2 or MCP3). The total scan time for the MCP joints was approximately 6 minutes, with an effective radiation dose of 8.4 μSv. The wrist scan covered 27.06 mm (330 slices). The first slice was located 2 mm distal to the distal margin of the radio-scaphoid joint, intersecting the lateral edge of the joint surface of the scaphoid (Fig. 1). The total scan time for the wrist was approximately 9 min, while the effective dose was 12.6 μSv. All scans were monitored for motion artifacts, and the scan was repeated once if the image grade exceeded 2 on the standard 5-point image quality scale [19].

#### HR-pQCT image analysis

All HR-pQCT images were analyzed by a rheumatologist (TS, HJC). Intra-reader and inter-reader reproducibility was assessed in both MCP joints and the distal radius of 14 patients. We calculated intraobserver reproducibility in the bone erosion analysis on the basis of five consecutive measurements, and calculated interobserver reliability from the measurements by three independent readers (two board-certified rheumatologists (TS, HJC) and a board-certified radiologist (JSG)). Radiographs were analyzed jointly by a musculoskeletal radiologist (TML) and two rheumatologists (TS and HJC), and MRI images were analyzed jointly by two radiologists (NC, TML). Standard HR-pQCT bone measurements at the distal radius, including BMD, cortical or trabecular bone microarchitecture were calculated semi-automatically (TS, MT) [20]. Analogous bone microarchitectural parameters were calculated in the distal head of the second and third metacarpal (MCH2 and MCH3). The analyzed region of interest was scaled in the slice-wise direction to span the distal 12% of the full metacarpal length as validated in a previous study [21]. The length of each metacarpal was measured (TS, HJC) on the baseline radiographs of the ipsilateral hand and used to normalize BMD and cortical thickness data in each bone. Three-dimensional measures of mean joint-space width (JSW) and volume were measured using an automated pipeline developed in our group [22]. All readers were blinded to the clinical information and there was a time interval of at least 4 weeks between the readings derived from the different imaging modalities.

Bone erosions in the MCP were evaluated from the HR-pQCT images following the recommended definitions and procedures developed by the Study GrouP for XTrEme-CT in Rheumatoid Arthritis (SPECTRA) [23]. We adapted analogous definitions and procedures for evaluating erosions at the wrist. An erosion was defined as a sharply demarcated cortical break spanning two or more consecutive axial slices and confirmed in the sagittal or coronal planes. If multiple erosions were present in the same quadrant (palmar, dorsal, radial or ulnar quadrant), the biggest erosion was measured. The maximum width and depth of erosions at the second and third MCH and phalangeal base (PB), and radial head were measured. Erosion volume was estimated using an ellipsoid model:

$$ {\mathrm{V}}_{\mathrm{erosion}}=\frac{4}{3}\uppi \times \frac{width}{2}\times depth\times \frac{perpendicular\kern0.34em width}{2} $$

Total erosion volume was calculated as the summation of all individual erosion volumes from MCH2, MCH3, PB2, PB3 and radial head. All erosion measurements and metacarpal lengths were performed using the open-source digital imaging and communications in medicine (DICOM) viewer Osirix V 7.0 (Nema, Rosslyn, VA, USA).

#### Radiographs and MRI and scoring

Bilateral dorsopalmar and oblique hand and feet radiographs were obtained at baseline. The bilateral radiographs were further evaluated using the well-known, modified Sharp/van der Heijde score for radiographs [24, 25].

MRI scans of the dominant wrist joints were acquired on a 3-T MR unit (MR 750 Wide Bore; GE Healthcare, Milwaukee, WI, USA) with an eight-channel phased array wrist coil (In vivo, Gainesville, FL, USA). Patients were examined in a supine position with their arm resting on the side of the body. To assess the bone marrow edema (BME) pattern, coronal T2-weighted iterative decomposition of water and fat with echo asymmetry and least-squares estimation (IDEAL) fast spin echo (FSE) water images (repetition time (TR)/echo time (TE) = 3500/50 ms, in-plane resolution = 0.2 mm, slice thickness = 2 mm) were performed. Coronal T1-weighted IDEAL spoiled gradient echo (SPGR) images (TR/TE = 15.3/2.9 ms, in-plane resolution = 0.2 mm, slice thickness = 1 mm) served for scoring erosion and joint-space narrowing. Coronal T1-weighted IDEAL FSE images (TR/TE = 600/9.9 ms, in-plane resolution = 0.44 mm, slice thickness = 2 mm) pre and post gadolinium injection were used to assess synovitis and erosions. MRI images were evaluated using the Outcome Measures in Rheumatology (OMERACT) RA-MRI scoring (RAMRIS) system to assess erosion, BME pattern, joint-space narrowing and synovitis [26, 27].

#### Statistical analysis

The paired *t* test was performed to compare patient characteristics and bone measurements within groups from baseline to 3 months. For intra-reader and inter-reader reproducibility of erosion assessment, Cohen’s kappa and coefficient variance (CV %) were calculated. Additionally, the least significant change (LSC) at 95% confidence level was calculated from CV%; *p* values <0.05 were considered statistically significant. All statistical analyses were performed using PASW Statistics ver. 18.0 (IBM Co., Armonk, NY, USA).

## Results

### Clinical results

Twenty-seven patients with clinically diagnosed RA were enrolled. There were 17 patients on anti-TNFα treatment combined with MTX treatment (anti-TNFα group) (6 with moderate, and 11 with high disease activity according to DAS-28-ESR at baseline), while 9 patients were on MTX treatment only (MTX-only group) (4 in remission and 5 with low disease activity). Table 1 summarizes the baseline characteristics of the two study groups. There were 9 patients in the MTX-only group and 14 in the anti-TNFα group who were followed up completely. Over the course of 3 months, among the patients on anti-TNFα treatment, six patients qualified as good responders, seven were moderate, and one was a non-responder, in accordance with the EULAR response criteria [16]. One patient could not be followed because of pregnancy and two other patients in the anti-TNFα group were lost to follow up for unknown reasons. No significant differences in demographics and baseline image measures were observed between the patients with and without follow up. In addition, over the 3-month treatment period, most of the clinical disease activity parameters significantly improved in the anti-TNFα group: both DAS-28-CRP and DAS-28-ESR exhibited significant decreases in this patient group (Table 2).

**Table 1:** Characteristics of patients with rheumatoid arthritis - combined or by group - at baseline

	All patients (*n* = 27)	MTX-only group (*n* = 10)	MTX + anti-TNFa group (*n* = 17)
Age (years), mean ± SD	51.9 ± 15.4	58.6 ± 15.8	47.7 ± 13.8
Sex (female:male)	22:5	7:3	15:2
Ethnicity			
Hispanic or Latino	25	10	15
Native Hawaiian or other Pacific Islanders	1	0	1
African American	1	0	I
Disease duration (years), mean ± SD	6.5 ± 5.4	7.1 ± 5.2	5.7 ± 5.6
Body mass index (kg/m^2^), mean ± SD	28.3 ± 7.1	24.5 ± 5.5	30.1 ± 7.6
Seropositivity of rheumatoid factor	14	3	11
Anti-CCP antibody	19	7	12
Cumulative prednisone dose (mg)	4.5 ± 3.8	1.4 ± 2.2	4.9 ± 3.6

*MTX* methotrexate, *TNFa* Tumor necrosis factor alpha, *DAS-28* disease activity score in 28 joints, *CCP* citrullinatcd protein

**Table 2:** Clinical features and radiological imaging scores in each treatment group from baseline and 3 months follow up in all patients with rheumatoid arthritis who had a 3-month follow-up visit

Total (*n* = 22)	MTX only group (*n* = 9)				MTX + anti-TNFα group (*n* = 13)			
Mean ± SD	Baseline	3 Months	Changes	*p* value*	Baseline	3 Months	Changes	*p* value*
Disease activity								
Swollen joints	0.7 ± 1.0	1.6 ± 1.8	0.9 ± 1.8	0.184	14.5 ± 3.9	6.3 ± 4.8	-8.2 ± 6.6	**<0.001**
Tender joints	0.7 ± 1.1	1.0 ± 1.3	0.3 ± 1.2	0.438	9.3 ± 6.2	4.4 ± 9.5	-4.9 ± 9.9	0.086
Global assessment of patient	7.4 ± 15.3	16.3 ± 25.7	8.9 ± 19.6	0.210	62.2 ± 16.8	41.4 ± 31.2	-20.8 ± 26.6	**0.015**
Physician	11.7 ± 10.9	14.1 ± 12.6	2.4 ± 9.4	0.459	42.8 ± 10.7	26.4 ± 12.1	-16.4 ± 18.2	**0.005**
ESR unm h) (mm/h)	18.0 ± 22.3	21.0 ± I8.3	3.0 ± 10.9	0.435	32.2 ± 21.9	29.7 ± 21.6	-2.4 ± 12.9	0.490
CRP tmg/L) (mg/L)	2.1 ± 1.1	4.6 ± 3.5	2.4 ± 2.9	**0.034**	21.9 ± 28.1	10.4 ± 9.8	-11.5 ± 22.2	0.088
DAS-28-ESR	1.9 ± 0.9	2.6 ± 0.9	0.7 ± 1.2	0.106	5.7 ± 1.1	3.8 ± 1.2	-2.0 ± 0.9	**<0.001**
DAS-28-CRP	l.8 ± 0.7	2.4 ± 0.8	0.6 ± 0.6	**0.032**	5.4 ± 0.9	3.5 ± 1.0	-I.7 ± 1.1	**<0.001**
HAQ	0.5 ± 0.7	0.7 ± 0.8	0.2 ± 0.3	0.084	1.6 ± 0.7	1.0 ± 0.7	-0.6 ± 0.6	**0.001**
Modified SHARP score								
Total	4.7 ± 6.0	NA	NA		26.1 ± 42.0	NA	NA	
Erosion	1.6 ± 2.6				13.5 ± 26.8			
JSN	3.1 ± 4.9				12.5 ± 17.0			
RAMRIS score								
JSN	0.2 ± 0.4	0.3 ± 0.7	0.1 ± 0.3	0.347	5.9 ± 8.4	5.9 ± 8.5	0.0 ± 0.4	<1.000
Synovitis	3.2 ± 2.1	3.1 ± 2.1	-0.1 ± 0.9	0.598	4.9 ± 2.6	3.9 ± 2.3	-l.0 ± 2.1	0.126
Bone erosion	2.6 ± 2.2	2.6 ± 2.6	0.0 ± 0.9	<1.000	20.5 ± 33.4	21.2 ± 33.3	0.6 ± 1.2	0.171
Bone edema	4.9 ± 4.0	4.9 ± 3.6	0.0 ± 1.1	<1.000	9.7 ± 7.8	7.1 ± 6.3	-2.6 ± 3.8	0.105

*MTX* methotrexate, *TNFa* tumor necrosis factor alpha, *ESR* erythrocyte sedimentation rate, *CRP* C-reactive protein, *DAS-28* disease activity score 28, *HAQ* health assessment questionnaire score, *JSN* joint space narrowing, *NA* not applicable, *RAMRIS* Outcome Measures in Rheumatology (OMERACT) rheumatoid arthritis-magnetic resonance imaging scoring. *Paired *t* test

### Bone erosion analysis

Overall, 73 erosions were detected by HR-pQCT. In the anti-TNFα group, erosions were located on the MCP2 (*n* = 23), MCP3 (*n* = 13), and distal radius (*n* = 11); the distribution was 7, 4 and 15, respectively, in the MTX-only group. During the 3-month follow-up period, the number of erosions in the both groups did not change (Table 3). In the anti-TNFα group, mean erosion volume throughout the joints numerically decreased over the 3 months, being statistically significant at MCP3 (*p* = 0.014) in all patients, and at MCP2 and MCP3 (*p* = 0.048 and *p* = 0.014, respectively), if we excluded the one non-responder. For the MTX-only group, on the contrary, we observed a slight increase overall in mean erosion volume throughout the joints, most pronounced at MCP2 (*p* = 0.006) and MCP3 (*p* = 0.019) (Table 3).

**Table 3:** Comparison of erosion volume and bone parameters by high-resolution peripheral quantitative computed tomography between baseline and 3 months in each treatment group

Total (*n* = 19)	MTX-only group (*n* = 9)				MTX + anti-TNFα group (*n* = 13)			
Mean ± SD	Baseline	3 Months	Changes (A)	*p* value*	Baseline	3 Months	Changes (A)	*p* value*
MCP2								
Erosion number (*n*)	7	7			23	23		
Erosion volume (mm^3^)	6.7 ± 6.4	7.5 ± 6.4	0.9 ± 0.5	**0.006**	41.4 ± 65.5	37.6 ± 60.3	-3.8 ± 5.2	**0.048**
Joint space								
Width (mm)	1.81 ± 0.14	1.80 ± 0.13	-0.01 ± 0.07	0.705	1.87 ± 0.20	1.80 ± 0.15	-0.07 ± 0.09	**0.028**
Volume (mm^3^)	133.6 ± 17.3	134.6 ± 16.0	1.0 ± 5.5	0.606	125.4 ± 20.5	118.7 ± 17.0	-6.68 ± 7.08	0.001
MCH2 microarchitecture BMD (mg/cm3)								
Total	279.9 ± 34.2	277.4 ± 40.2	-6.2 ± 16.1	0.288	316.2 ± 34.7	327.8 ± 45.9	11.6 ± 33.0	0.268
Trabecular	236.2 ± 26.9	233.6 ± 33.2	-2.6 ± 20.4	0.731	259.0 ± 25.6	266.9 ± 41.0	7.8 ± 26.8	0.353
MCP3								
Erosion number (*n*)	4	4			13	13		
Erosion volume (mm3)	2.3 ± 11.5	3.1 ± 1.2	0.8 ± 0.5	**0.019**	21.6 ± 31.5	18.9 ± 29.0	-2.7 ± 2.5	**0.014**
Joint space								
Width (mm)	1.72 ± 0.14	1.74 ± 0.15	0.02 ± 0.05	0.226	1.83 ± 0.12	1.74 ± 0.14	-0.10 ± 0.07	0.001
Volume (mm^3^)	138.9 ± 17.0	142.1 ± 20.2	3.2 ± 4.3	0.069	131.9 ± 24.1	125.6 ± 21.4	-4.3 ± 4.6	**0.003**
MCH3 microarchitecture BMD (mg/cm^3^)								
Total	279.9 ± 28.0	280.8 ± 25.0	1.0 ± 7.0	0.706	324.5 ± 35.7	321.8 ± 34.8	-2.7 ± 8.4	0.298
Trabecular	238.9 ± 20.1	241.9 ± 18.1	3.0 ± 8.1	0.322	269.4 ± 26.7	267.6 ± 26.1	-1.7 ± 8.5	0.513
Distal radius								
Erosion number (*n*)	15	15			11	11		
Erosion volume (mm^3^)	21.1 ± 26.4	23.4 ± 29.1	1.6 ± 3.3	0.075	199.9 ± 362.6	188.4 ± 331.4	-15.5 ± 31.7	0.211
Joint space								
Width (mm^3^)	2.04 ± 0.24	2.04 ± 0.18	-0.01 ± 0.12	0.981	2.09 ± 0.29	2.11 ± 0.29	0.01 ± 0.17	0.758
Volume (mm^3^)	249.8 ± 29.3	261.1 ± 30.5	11.3 ± 22.3	0.197	247.5 ± 67.1	240.2 ± 71.2	-7.3 ± 25.0	0.356
Wrist microarchitecture BMD (mg/cm^3^)								
Total	272.3 ± 61.3	272.8 ± 60.3	0.6 ± 4.3	0.709	356.0 ± 58.6	354.0 ± 58.4	-2.0 ± 7.1	0.357
Cortical	793.7 ± 82.9	792.1 ± 83.2	-1.6 ± 9.8	0.663	873.4 ± 42.7	874.9 ± 43.7	1.5 ± 9.7	0.607
Trabecular	148.0 ± 31.0	148.6 ± 30.8	0.6 ± 2.7	0.543	174.5 ± 53.7	172.0 ± 51.2	-2.5 ± 5.1	0.115
Cortical thickness (μm)	607.8 ± 200.4	606.7 ± 199.5	3.8 ± 21.2	0.900	824.6 ± 142.7	830.8 ± 140.8	6.2 ± 27.3	0.450
Trabecular								
Number	1.82 ± 0.14	1.80 ± 0.17	-0.02 ± 0.15	0.748	1.97 ± 0.45	1.94 ± 0.42	-0.03 ± 0.10	0.315
Thickness (μm)	68.9 ± 12.6	69.9 ± 12.6	1.4 ± 5.0	0.441	72.9 ± 10.8	73.1 ± 10.2	0.2 ± 4.1	0.898
Separation (μm)	485.7 ± 51.1	491.2 ± 60.0	5.6 ± 45.6	0.740	471.5 ± 171.7	475.1 ± 156.3	3.6 ± 34.8	0.725

The distal radius joint space was measured at the radio-lunar joint. *MTX* methotrexate, *TNFa* tumor necrosis factor alpha, *MCH* metacarpal heads, *BMD* bone mineral density. *Paired *t* test. Bold characters mean *p *< 0.05

The kappa scores (*k*) for intra-reader and inter-reader reproducibility for detecting erosions were 0.970 (*p* < 0.001) and 0.818 (*p* = 0.001), respectively. For the measurement of erosion volume, intra-reader agreement was 3.43% (root mean square (RMS) %CV), and inter-reader agreement was 3.92% (RMS %CV). The volume changes of 17 erosions (out of 26 erosions) in the MTX-only group and 32 erosions (out of 47 erosions) in the anti-TNFα group, respectively, exceeded the least significant change (LSC).

### Joint space analysis

We next investigated changes in joint space geometry, namely in width and volume, from baseline to 3 months using a 3D joint space quantification derived from HR-pQCT images. In the anti-TNFα group, we consistently observed a decrease in joint space volume and joint space width throughout the measured joint sites over the 3-month follow-up period (Table 3). The opposite pattern was observed in the MTX only group, where we observed almost no change or a trending increase in joint volume over time.

### Microarchitecture analysis

Additionally, we investigated other HR-pQCT measurements in our study to assess changes in bone microarchitecture at the MCP and distal radius. There were no differences in bone microarchitecture from baseline to 3 months with either of the treatments.

### Correlations of parameters evaluated by HR-pQCT

**Fig. 2:**
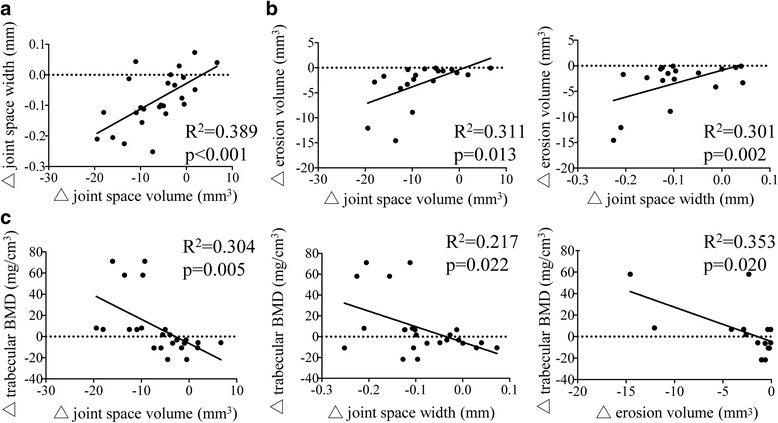
**a** Pearson correlations between joint space volume and joint space in the metacarpal bone imaged by high-resolution peripheral quantitative computed tomography (HR-pQCT) in the anti-TNFα treatment group. **b** Pearson correlation between changes in erosion volume and joint space in the metacarpal bone imaged by HR-pQCT in the anti-TNFα treatment group. **c** Pearson correlation between changes in trabecular bone mineral density (BMD), and changes in erosion volume and joint space in the metacarpal bone imaged by HR-pQCT in the anti-TNFα treatment group

To better understand the 3-month changes in bone microstructure induced by anti-TNFα treatment, we assessed the correlation between changes in joint space, in bone erosion and in bone microarchitecture in the anti-TNFα group (Fig. 2). At the MCP joints, there was strong significant correlation between changes in joint space width and volume as expected (Fig. 2a). Changes in bone erosion volume were significantly correlated with changes in joint space width and volume (Fig. 2b). Changes in total and trabecular BMD were significantly negatively correlated with changes in erosion volume, joint space width and volume (Fig. 2c). The other parameters did not demonstrate significant correlation. In the wrist joint, none of the parameters were statistically significantly correlated (data not shown).

**Fig. 3:**
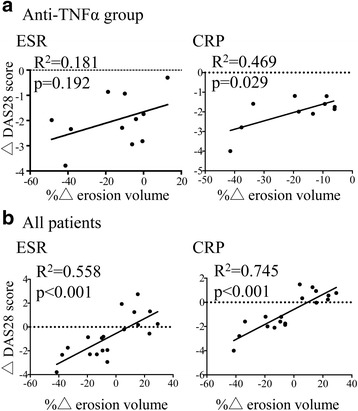
**a** Pearson correlation between percentage change in erosion volume and change in the disease activity score in 28 joints (DAS-28) in the anti-TNFα treatment group. **b** Pearson correlation between percentage change in erosion volume and change in the DAS-28 score in all patients

Next we investigated correlation between changes in bone erosion volume and clinical status. Because erosion volume and change varied between MCP and wrist joints, we calculated percentage changes in the total bone erosion of the MCP and wrist joint from baseline to 3 months and investigated correlation with the change in the DAS-28 ESR and CRP (Fig. 3). In the anti-TNFα group, the percentage change in total erosion volume from baseline to 3 months was positively correlated, albeit statistically insignificant, with change in the DAS-28 ESR and CRP (Fig. 3a). In all patients, percentage change in total erosion volume from baseline to 3 months was statistically significantly and positively correlated with change in the DAS-28 ESR and CRP (Fig. 3b).

### Radiography and MRI results

Modified Sharp scores were 4.7 ± 6.0 in the MTX group vs. 26.1 ± 42.0 in the anti-TNFα group at baseline. The RAMRIS bone edema score decreased, albeit this was statistically insignificant, in the anti-TNFα group from baseline to 3 months. However, there were no differences in the other RAMRIS scores, including bone erosion score from baseline to 3 months, in either group (Table 2). No significant correlation was observed between change in HR-pQCT parameters and the RAMRIS scores.

## Discussion

To the best of our knowledge, this is the first HR-pQCT data characterizing and quantifying the early (3-month) effect of anti-TNFα treatment on bone erosion progression and bone microstructure in RA. In accordance with a significant decrease in the clinical DAS-28 scores in the anti-TNFα treatment group, erosion volumes and the joint space tended to decrease, especially in the MCP joints, suggesting that the bone changes are well-reflected in the clinical response. To date, there has only been a single retrospective HR-pQCT study reporting a significant reduction in erosion depth in patients with RA treated with anti-TNFα for one year [28]. Our data showed a tendency toward decreased erosion volume even though patients were on anti-TNFα treatment for only 3 months; in this regard, our results confirm and expand on the previous report [28]. Additionally, our findings are consistent with previous studies that showed anti-TNFα treatments prevent erosion progression compared to treatment with MTX alone [4–7], although we showed it as early as 3 months, while previous studies usually utilized 1-year-old to 2-year-old radiographic findings. Therefore, the high sensitivity of HR-pQCT could potentially help to reduce the length of future clinical trials.

**Fig. 4:**
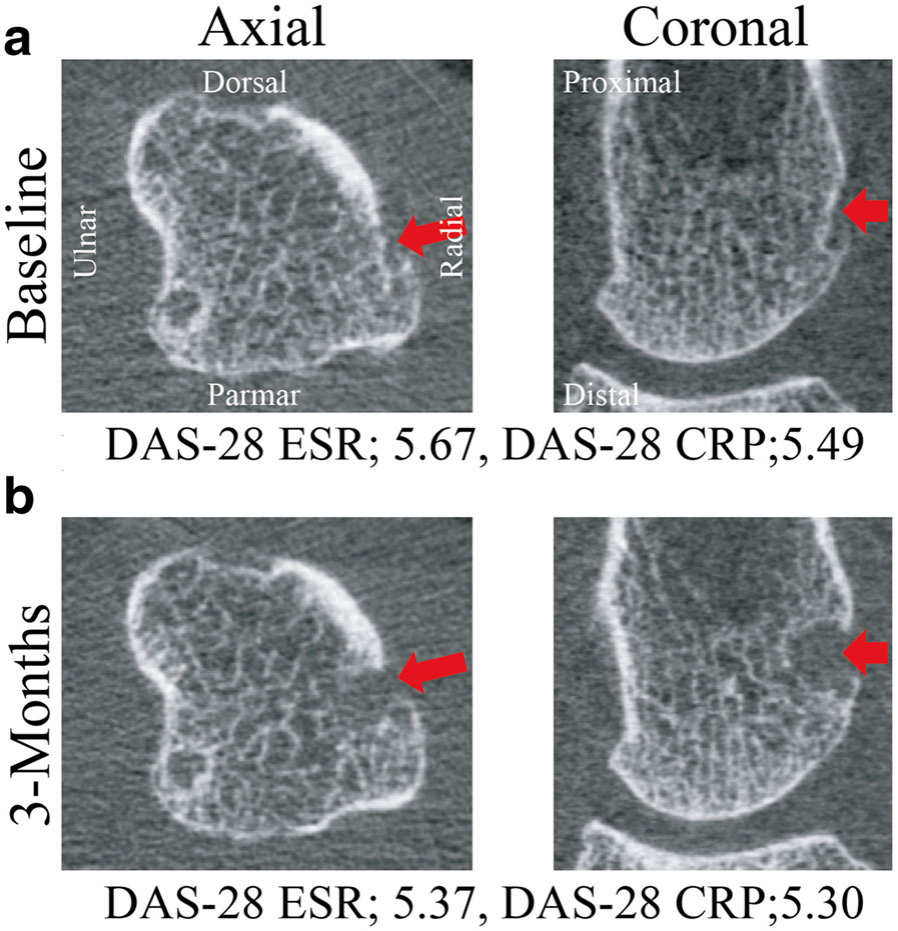
Axial and coronal high-resolution peripheral quantitative computed tomography (HR-pQCT) images at the second metacarpal head in a patient who demonstrated increased erosion (erosion volume = 3.90 mm^3^ at baseline (**a**) and 12.38 mm^3^ at 3 months (**b**)). Red arrows indicate bone erosion. DAS-28 disease activity score in 28 joints

Interestingly, even though the anti-TNFα group had higher disease activity than the MTX-only group at both baseline and 3 months, the anti-TNFα treatment seems to have prevented an increase in bone erosion in this group. This could for example be explained by the hypothesis that anti-TNFα might not only have suppressed inflammation but also osteclastogenesis, which is one of the major pathologic steps in RA pathogenesis [1], and the RANK-RANKL complex is a principal regulator and is activated in the early phase of osteoclast differentiation [29]. TNFα is a major proinflammatory cytokine in RA and is reported to influence osteoclast precursor cells through the expression or the activation of RANK [30]. Certainly, considered with the significant positive correlation between change in erosion volume and the DAS-28 scores, control of disease activity is thought to be important to prevent bone destruction, and this is supported by our observation of increased erosion volume in a patient who was not responding clinically to the anti-TNF treatment (Fig. 4). However, a recent randomized controlled trial showed that denosumab, a RANKL inhibitor, decreases bone erosion volume within 6 months in patients with moderately controlled RA, but not within 3 months [31]. Therefore, our findings suggest that anti-TNFα could have an even earlier effect in preventing and restoring arthritis-induced bone destruction if patients respond to the therapy.

The changes in erosion volume were correlated with the changes in joint space and trabecular BMD of the MCP joints from baseline to 3 months in the anti-TNFα group. Although joint space narrowing is thought to be one of the typical radiographic characteristics in patients with RA [32], interestingly the anti-TNFα group had a significant decrease in joint space volume at the MCP joints following clinical response to therapy. It could be explained by the reduced amount of effusion and inflammation in the joint following anti-TNFα treatment in this cohort. However, the joint space width in the MTX-only group was smaller than in the anti-TNF group. The reason was thought to be that the anti-TNF group included some patients with subluxation, who had wider joint spaces. Therefore, the joint space changes may not be linear in RA, depending on the stage of disease and treatment. We also found that a decrease in erosion volume was correlated with an increase in trabecular BMD and structures, consistent with an overall bone damage prevention effect with anti-TNF treatment, as discussed previously.

Although we observed moderate to strong correlation between changes in erosions and changes in joint space and trabecular BMD in the MCP joints, we did not identify similar correlation for the wrist joints. However, the wrist joint is more complicated, including radio-lunate, radio-scaphoid and radio-ulnar joints, and is wider than the MCP joints, so it might be more difficult to capture rapid changes in bone destruction. Additionally, the wrist joints are much more dependent than the MCP joints on abduction and adduction effects, as the wrist joint allows joint movement in two planes, which are not possible in the hinged (ginglymus) MCP joints which can only be moved in one plane.

Of note, our data did not show any significant correlation between HR-pQCT erosion volume changes and changes in MR-based RAMRIS scores. This suggests that MRI and HR-pQCT are two distinct imaging modalities with different strengths and focus to evaluate RA pathophysiology. MRI measures, especially synovitis and BME have been suggested to be indicators of inflammation within the joints in RA. However, in terms of RAMRIS synovitis and BME scores, the range of scoring is too small (0–3) to show significant correlation with the changes in HR-pQCT parameters in this study. Therefore, more sensitive quantitative MRI analysis such as synovitis volume and BME-like lesion volume might be necessary for comparison with these HR-pQCT-driven parameters. On the other hand, in terms of bone erosion, a recent study reported that MRI has lower sensitivity in the detection of bone erosions (60%) and detection of osteosclerosis (24%) compared to HR-pQCT in the MCP2, MCP3 and wrists of patients with RA [33]. Additionally, a recent systemic review reported that RAMRIS erosions and joint-space narrowing scores could not detect early significant changes [34]. Therefore, focusing on bone-related changes, HR-pQCT is a powerful tool for providing objective and sensitive measures of bone erosions, cortical and trabecular bone structure and joint space morphology. These HR-pQCT-derived imaging markers are promising outcome measures for future potential trials that combine treatment of inflammation and bone damage (anti-absorption treatment, for example) in RA.

There are some limitations of this study to report: first, HR-pQCT has a limited field of view (FOV), as compared to other imaging modalities such as MRI, whole body CT or cone-beam radiographic imaging systems, which are also more widely available than HR-pQCT. Furthermore, HR-pQCT is normally limited to a small number of joints, primarily due to keeping the overall scan within a clinically feasible time. In future studies, mid-carpal joint and more distal regions may be considered as well. Second, this study was limited by its small sample size, but this is the first report about applying HR-pQCT at 3 months after anti-TNFα treatment and was meant to be exploratory in nature. In the future, larger cohort studies are warranted to confirm the findings from this study. Third, the study was also limited by a follow up of 3 months. Although we targeted the early changes in bone during treatment with anti-TNFα in this study, we are currently following up these patients at 1 year after treatment to investigate long-term bone changes using HR-pQCT. Finally, bone turnover markers were not checked in this study. Bone markers e.g. tartrate-resistant acid phosphatase 5b (TRAP5b) have been shown to correlate with bone erosions in patients with RA imaged by HR-pQCT [35]. Although there are some risk factors that influence bone turnover markers in patients with RA, such as steroid use and immobility, it should be addressed in future studies as to whether bone markers are useful in predicting changes in erosion volume in patients with RA who are receiving anti-TNFα.

## Conclusions

Using HR-pQCT we demonstrated that anti-TNFα treatment can prevent an increase in bone erosion within a 3-month treatment period, consistent with a significant decrease in disease activity. Our results suggest that HR-pQCT is a sensitive and powerful tool for quantifying bone changes and monitoring RA treatment even within a short-term time window such as 3 months. However, future larger HR-pQCT studies with follow-up periods of 3 months or less are needed to validate these initial observations.

## Abbreviations

BMD: Bone mineral density
BME: Bone marrow edema
CRP: C-reactive protein
CT: Computed tomography
CV: Coefficient variance
DAS: Disease activity score
DICOM: Digital imaging and communications in medicine
ESR: Erythrocyte sedimentation rate
FSE: Fast spin echo
HR-pQCT: High-resolution peripheral quantitative computed tomography
IDEAL: Iterative decomposition of water and fat with echo asymmetry and least-squares estimation
IL: Interleukin
JSW: Joint space width
LSC: Least significant change
MCH: Distal head of the metacarpal
MCP: Metacarpophalangeal
MRI: Magnetic resonance imaging
MTX: Methotrexate
PB: Phalangeal base
RA: Rheumatoid arthritis
RAMRIS: OMERACT Outcome Measures in Rheumatology (OMERACT) rheumatoid arthritis-magnetic resonance imaging scoring
SPGR: Spoiled gradient echo
TE: Echo time
TNFα: Tumor necrosis factor alpha
TR: Repetition time
